# TRPM7 maintains progenitor-like features of neuroblastoma cells: implications for metastasis formation

**DOI:** 10.18632/oncotarget.3315

**Published:** 2015-03-05

**Authors:** Jeroen Middelbeek, Daan Visser, Linda Henneman, Alwin Kamermans, Arthur J. Kuipers, Peter M. Hoogerbrugge, Kees Jalink, Frank N. van Leeuwen

**Affiliations:** ^1^ Laboratory of Pediatric Oncology, Radboud Institute for Molecular Life Sciences, Radboudumc, Nijmegen, The Netherlands; ^2^ Division of Cell Biology, The Netherlands Cancer Institute, Amsterdam, The Netherlands; ^3^ Princes Maxima Center for Pediatric Oncology, Utrecht, The Netherlands

**Keywords:** neuroblastoma, trpm7, metastasis, differentiation, snai2

## Abstract

Neuroblastoma is an embryonal tumor derived from poorly differentiated neural crest cells. Current research is aimed at identifying the molecular mechanisms that maintain the progenitor state of neuroblastoma cells and to develop novel therapeutic strategies that induce neuroblastoma cell differentiation. Mechanisms controlling neural crest development are typically dysregulated during neuroblastoma progression, and provide an appealing starting point for drug target discovery.

Transcriptional programs involved in neural crest development act as a context dependent gene regulatory network. In addition to BMP, Wnt and Notch signaling, activation of developmental gene expression programs depends on the physical characteristics of the tissue microenvironment. TRPM7, a mechanically regulated TRP channel with kinase activity, was previously found essential for embryogenesis and the maintenance of undifferentiated neural crest progenitors. Hence, we hypothesized that TRPM7 may preserve progenitor-like, metastatic features of neuroblastoma cells.

Using multiple neuroblastoma cell models, we demonstrate that TRPM7 expression closely associates with the migratory and metastatic properties of neuroblastoma cells *in vitro* and *in vivo*. Moreover, microarray-based expression profiling on control and TRPM7 shRNA transduced neuroblastoma cells indicates that TRPM7 controls a developmental transcriptional program involving the transcription factor SNAI2. Overall, our data indicate that TRPM7 contributes to neuroblastoma progression by maintaining progenitor-like features.

## INTRODUCTION

Neuroblastoma is an embryonic tumor derived from cells of the neural crest. Survival rates are excellent for patients with low- and intermediate-risk neuroblastomas. In contrast, patients with high-risk neuroblastomas present with metastatic disease at diagnosis and require intensive treatment regimens. Although virtually all patients initially respond to treatment, a therapy resistant pool of poorly differentiated cells may arise that leads to refractory disease for which no treatment options are currently available [1, 2]. To develop more effective treatment strategies for these patients, there is an urgent need to understand the molecular mechanisms that control the progenitor state of neuroblastoma cells. The similarities between neural crest development and neuroblastoma progression have previously been recognized [3–5] and provide an appealing starting point for drug target discovery.

The neural crest is a population of cells that arises at the borders of the neuroectoderm during early embryogenesis, spreads to different parts of the embryo and gives rise to a multitude of cell types, including the peripheral sympathetic neurons [6]. Mechanisms controlling migration and differentiation of neural crest cells are typically dysregulated during neuroblastoma development and progression. For instance genes that maintain the balance between proliferation and differentiation of neural crest cells, such as *MYCN*, *ALK* and *PHOX2B*, are often found mutated or overexpressed in high-risk neuroblastomas [3, 4]. Additionally, metastatic neuroblastoma cells adopt a pro-migratory developmental program known as epithelial-to-mesenchymal transition (EMT), which allows neural crest cells to delaminate from the neural plate borders and spread throughout the embryo [7–10]. Consistently, *in vitro* evidence indicates that genes involved in EMT of neural crest cells, including transcription factors such as SNAI2, are misregulated in metastatic neuroblastomas [4, 10, 11].

BMP, Wnt and Notch mediated signal transduction pathways act in concert to control neural crest formation, migration and maturation [6]. Additionally, mechanical input from the cellular environment drives neural crest maturation [12–16]. As these signals are essential for proper tissue development and maintenance of cellular quiescence, perturbed mechanical signaling can propagate de-differentiation, uncontrolled cell proliferation, tissue invasion and therapy resistance in solid tumors, including neuroblastoma [17–29]. Members of the mammalian Transient Receptor Potential (TRP) cation channel family are considered key regulators of the mechanical interactions between the cell and its microenvironment. Tethered to the cytoskeleton, their ion conducting properties can be modulated by different stimuli, including mechanical cues, resulting in responses that range from adhesion remodeling to cellular differentiation [30–32]. Indeed, we showed that TRPM7, a TRP-cation channel with kinase activity, directly interacts with the actomyosin cytoskeleton and controls cell-matrix interactions in breast cancer cells as well as in neuroblastoma cells [33, 34]. Moreover, we and others showed that TRPM7 functionally contributes to the progression of a number of malignancies *in vitro* and *in vivo* (reviewed in [35]). However, the mechanisms by which TRPM7 drives tumor progression remain poorly understood.

Studies using TRPM7 conditional knockouts demonstrate that TRPM7 expression is required during early stages of embryogenesis [36, 37]. Moreover, TRPM7 appears to be essential for the maintenance of multi-potent neural crest cells [37]. Hence, we hypothesize that TRPM7 expression and/or activity may contribute to neuroblastoma progression by disrupting normal neural crest cell maturation and preserving progenitor-like features in tumor cells. Consistent with this notion, we show here that TRPM7 overexpression confers a metastatic phenotype onto an otherwise poorly metastatic neuroblastoma cell line, while shRNA-mediated knockdown of TRPM7 reduces the migratory properties of neuroblastoma cells. In addition, by gene expression profiling we demonstrate that TRPM7 is required for the maintenance of a progenitor-like gene expression program in human neuroblastoma cell lines.

## RESULTS

### TRPM7 confers a malignant phenotype onto poorly metastatic neuroblastoma cells

**Figure 1:**
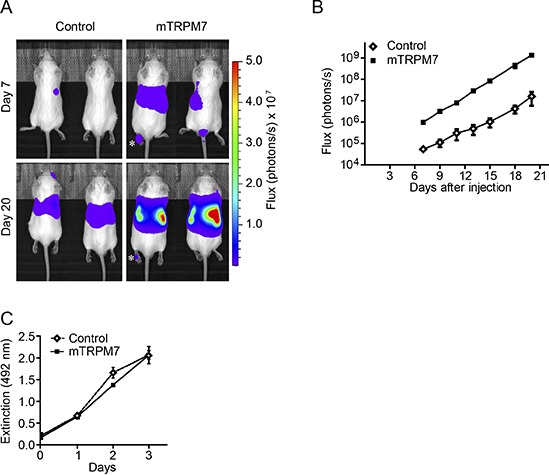
TRPM7 increases the metastatic potential of N1E-115 cells **(A)** Representative bioluminescent images of mice, 7 and 20 days after intravenous injections with N1E-115 control or mTRPM7 overexpressing cells (*n* = 9 mice in each group). Photon fluxes are set to the same scale (photons / s). Asterix indicates an example of bioluminescence observed in a limb. **(B)** Quantification of bioluminescence in the abdominal region between day 7 and day 20 post-injection. Data are mean ± SEM of *n* = 9 mice per group. Data presented are from 1 out of 2 independent experiments that show highly reproducible results. **(C)** Quantification of cell proliferation, determined by MTS assays. Data are mean extinction at 492 nm ± SEM of *n* = 3 experiments.

To address if TRPM7 contributes to the malignant properties of neuroblastoma cells, we assessed whether TRPM7 overexpression promotes metastasis formation of poorly metastatic murine N1E-115 neuroblastoma cells *in vivo*. To this end, we intravenously injected luciferase expressing neuroblastoma cells that were previously generated to either overexpress mouse TRPM7 (mTRPM7) or an empty vector control (Control) (Supplementary Figure S1A) [33], into *Rag2^−/−^Il2rg^−/−^* immunodeficient mice. Non-invasive bioluminescence imaging was used to monitor tumor cell dissemination and growth. Bioluminescence signals were observed at day 7 post-injection and progressively increased over time (Figure 1A & 1B), showing that injected cells survived, proliferated and formed metastasis. In good agreement with earlier reports on metastasis of neuroblastoma cells in mice [38], bioluminescence originated predominantly from the abdominal region. Strikingly, the abdominal signal in N1E-115 mTRPM7 injected mice was much higher at all time points (day 7: control = 5.41 × 10^4^ ± 9.92 × 10^3^ photons/s, *n* = 9; mTRPM7 = 9.75 × 10^5^ ± 1.63 × 10^5^ photons/s, *n* = 9). Note that the progressive increase in bioluminescence was comparable in both groups, suggesting that the *in vivo* proliferation rate of neuroblastoma cells was not affected by TRPM7 expression levels (Figure 1B). Indeed, MTS assays indicated that mTRPM7 overexpression did not affect *in vitro* proliferation rates (Figure 1C).

### TRPM7 promotes metastatic spread to liver and bone marrow, but not *in vivo* proliferation

**Figure 2:**
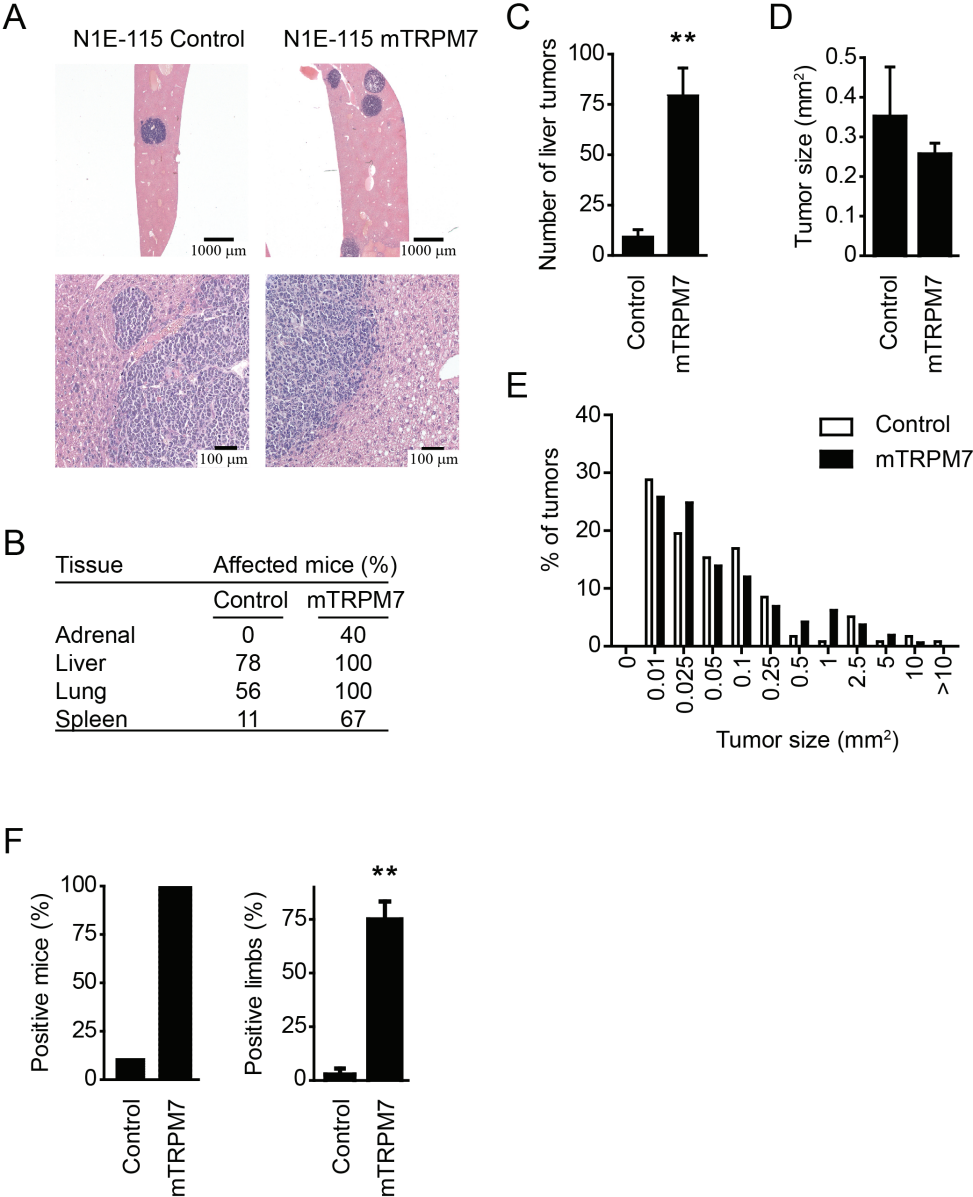
TRPM7 promotes metastatic spread to liver and bone marrow **(A)** Representative H&E staining on liver tissue, collected 20 days after injection with N1E-115 cells. Prominent tumors in the liver indicate that both N1E-115 control and mTRPM7 overexpressing cells metastasize to the liver. **(B)** Histopathological analysis of mouse tissue sections, collected 20 days after injection with either N1E-115 control or mTRPM7 overexpressing cells. Mouse tissues were scored for the presence of tumor cells. Adrenal glands of 5 mice were scored for presence of tumor cells, whereas 9 mice were dissected for other tissues. **(C)** Quantification of the number of liver tumors per mouse, measured in resected liver tissue from mice injected with N1E-115 control or mTRPM7 overexpressing cells. Data are mean ± SEM of *n* = 9 mice in each group. **p < 0.01, two-tailed unpaired *t*-test with Welch correction. **(D)** Quantification of mean liver tumor size in mice injected with N1E-115 control and mTRPM7 overexpressing cells. Data are mean ± SEM of *n* = 9 mice in each group. **(E)** Size distribution of tumors in liver sections emphasizes similar growth rates for both cell lines. **(F)** Percentage of mice (left panel) and limbs (right panel) with bone marrow metastases. Data are mean ± SEM of *n* = 9 mice in each group. **p < 0.01, two-tailed unpaired *t*-test with Welch correction.

As the proliferation rate of N1E-115 mTRPM7 cells did not differ from control cells *in vitro*, the increased abdominal signals of mice injected with these cells suggest that dissemination is more widespread. Histopathological analysis showed that the dissemination pattern was similar in both groups, with metastases predominantly present in the liver (Figure 2A & 2B). However, image analysis of liver paraffin sections demonstrated that much more tumors were present in mice injected with N1E-115 mTRPM7 cells (control = 9.5 ± 3.3 tumors per liver section; mTRPM7 = 79.7 ± 13.4 tumors per liver section; *p* < 0.01, *n* = 9) (Figure 2A & 2C). Consistent with the observation that TRPM7 does not affect proliferation *in vitro*, mTRPM7 overexpression had no effect on mean tumor size (control = 0.35 ± 0.13 mm^2^, *n* = 118 tumors; mTRPM7 = 0.26 ± 0.03 mm^2^, *p* = 0.23, *n* = 786 tumors) (Figure 2D & 2E).

In addition to the liver, bone and bone marrow metastases are commonly observed in neuroblastoma patients [39]. We therefore isolated bone marrow content from each limb and analyzed luciferase activity. Limbs were considered positive when bioluminescence was five times above background. Strikingly, only one limb from a single N1E-115 control injected mouse met this criterion, whereas all mice injected with N1E-115 mTRPM7 cells scored positive with an average of 3 affected legs per mouse (control = 1 limb in 1 out of 9 mice; mTRPM7 = 9 out of 9 mice affected with 27 out of 36 limbs scoring positive, *p* < 0.01) (Figure 2F). Hence, we conclude that TRPM7 enhances the metastatic potential, but not proliferation rate, of N1E-115 neuroblastoma cells *in vivo*.

### TRPM7 expression does not affect viability and proliferation of human neuroblastoma cells

Although TRPM7 overexpression did not affect proliferation of mouse N1E-115 neuroblastoma cells, others have suggested a role for TRPM7 in neuroblastoma cell proliferation, specifically in a MYCN-amplified context [40]. SH-EP2 is a non-neuronal (S-type) subclone of the SK-N-SH human neuroblastoma cell line with no expression of MYCN. In turn, MYCN is amplified in the neuroblastic (N-type) subclone of SK-N-SH cells, named SH-SY5Y [41]. As MYCN was essential for proliferation of these cells [42], the SH-SY5Y neuroblastoma model provides a physiological relevant model to study the functional interactions between TRPM7 and MYCN in cell proliferation.

**Figure 3:**
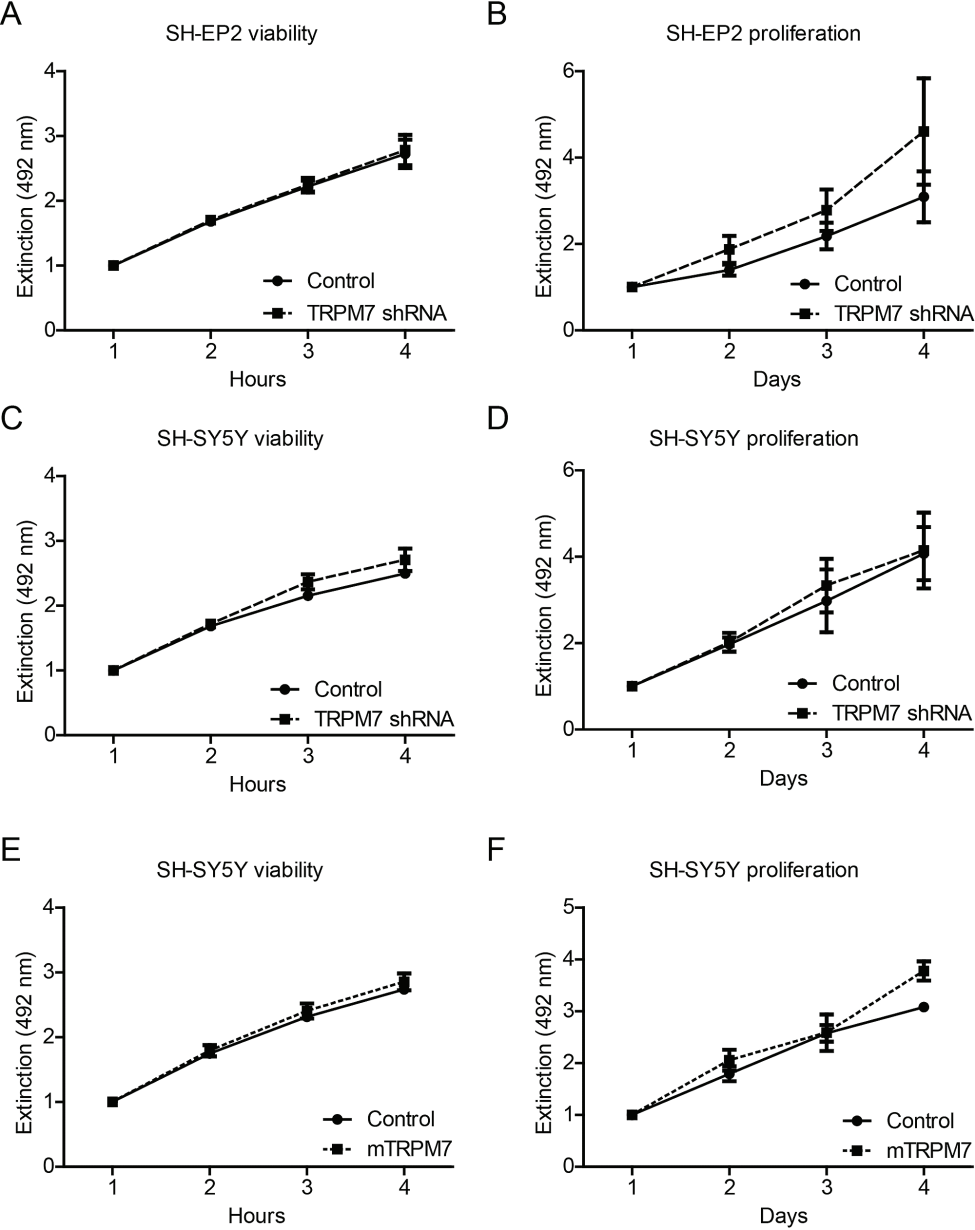
Manipulation of TRPM7 expression does not affect neuroblastoma cell viability and proliferation Quantification of cell viability and proliferation, determined by MTS assays. Viability was assessed over 4 hours, proliferation was assessed over 4 days. Data represents normalized mean extinction at 492 nm mean ± SEM of *n* > 2 experiments performed in triplo. **(A–D)** Effects of TRPM7 shRNA on viability and proliferation of SH-EP2 cells and SH-SH-5Y human neuroblastoma cells. **(E & F)** Effects of mTRPM7 overexpression on viability and proliferation of SH-SY5Y human neuroblastoma cells.

To see if and how TRPM7 affects viability and proliferation of MYCN-negative and positive neuroblastoma cells, we generated stable TRPM7 knockdown SH-EP2 and SH-SY5Y cells using a previously characterized lentiviral shRNA construct [34] (Supplementary Figure S1B & S1D). TRPM7 knockdown did not markedly affect viability and proliferation of MYCN-negative SH-EP2 cells, as we assessed by MTS-assays (Figure 3A & 3B, Supplementary Figure S2A & S2B). In contrast to previous reports [40], however, stable knockdown of TRPM7 did not affect viability and proliferation of MYCN-amplified SH-SY5Y cells (Figure 3C & 3D). To exclude the possibility that endogenous TRPM7 expression levels were limiting MYCN-induced proliferation, we overexpressed mTRPM7 in SH-SY5Y cells (Supplementary Figure S1C) and subsequently assayed viability and proliferation. Again, no effects of TRPM7 expression levels were observed (Figure 3E & 3F). From these experiments, we conclude that in our hands, stable knockdown or overexpression of TRPM7 does not affect viability and proliferation of both MYCN-negative and MYCN-amplified neuroblastoma cell lines.

### TRPM7 promotes neuroblastoma cell migration

**Figure 4:**
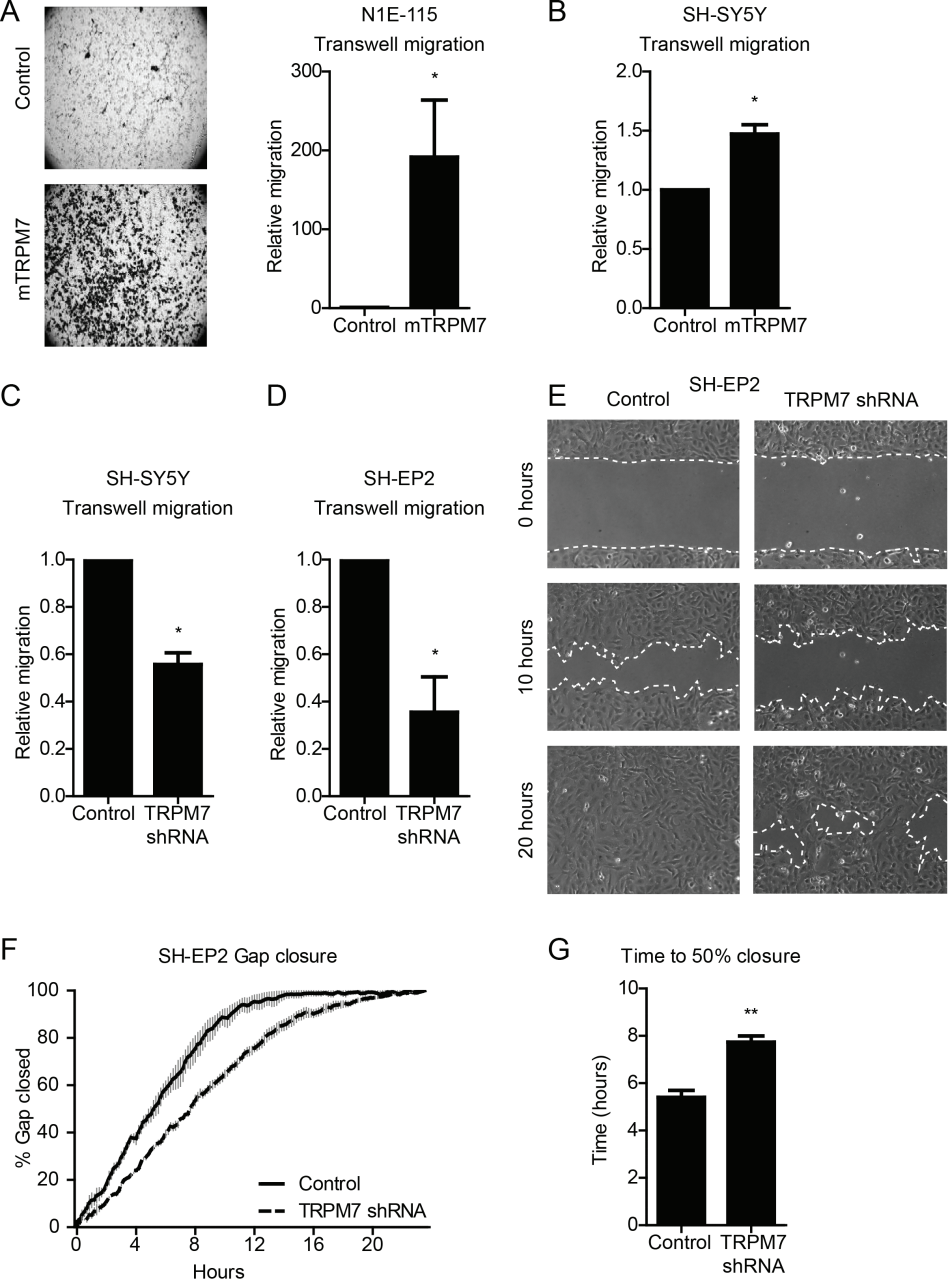
TRPM7 drives mouse and human neuroblastoma cell migration **(A)** & **(B)** Transwell migration of N1E-115 (*n* = 6) and SH-SY5Y (*n* = 3) control and mTRPM7 overexpressing cells. Equal numbers of cells were allowed to migrate towards a serum gradient for 48 hours. Data are normalized to control and represent mean ± SEM of *n* > 3 independent experiments performed in duplicate. **(C)** & **(D)** Transwell migration of SH-SH5Y and SH-EP2 control and TRPM7 shRNA cells. Equal numbers of SH-SY5Y and SH-EP2 cells were allowed to migrate towards a serum gradient for 48 and 24 hours respectively. Data are normalized to control and represent mean ± SEM of *n* = 3 independent experiments performed in duplicate. **(E)** Representative images of gap closure by SH-EP2 control and TRPM7 shRNA cells at time points 0, 10 and 20 hours. **(F)** Gap closure over time, presented as percentage of gap size at time point 0 hours. **(G)** Quantification of time to 50% gap closure. Data in (F) and (G) represent mean from *n* = 3 independent experiments, each performed in duplicate. *p < 0.05, **p < 0.01, two-tailed unpaired *t*-test.

We previously showed that TRPM7 is required for breast cancer cell migration [34]. Since metastatic neuroblastoma cells adopt the migratory potential of disseminating neural crest cells, we set out to evaluate the effects of TRPM7 expression on neuroblastoma cell migration. We first compared the migratory properties of N1E-115 control and mTRPM7 cells by assaying migration towards a serum gradient in Boyden chambers. Whereas control cells remained immobile during 48 hours, mTRPM7 overexpression enhanced the ability of neuroblastoma cells to cross the transwell membrane (191.5 ± 72 fold mTRPM7 cells relative to control cells, *p* = 0.05, *n* = 6) (Figure 4A). Similarly, mTRPM7 overexpression promoted transwell migration of human SH-SY5Y cells (1.5 ± 0.078 fold mTRPM7 cells relative to control cells, *p* = 0.026, *n* = 3) (Figure 4B). In reciprocal experiments, TRPM7 knockdown limited serum induced transwell migration of SH-SY5Y cells (0.56 ± 0.049 fold TRPM7 shRNA cells relative to control cells, *p* = 0.012, *n* = 3), as well as SH-EP2 cells (0.36 ± 0.15 fold TRPM7 shRNA cells relative to control cells, *p* = 0.049, *n* = 3) (Figure 4C & 4D). The substrate adhesive characteristics of SH-EP2 cells allowed us to assess cell migration in gap closure assays. Consistent with serum induced transwell migration, reduced TRPM7 expression levels significantly reduced gap closure speed (control = 5.4 ± 0.3 hrs; TRPM7 shRNA = 7.7 ± 0.26 hrs to 50% closure, *p* = 0.005, *n* = 3) (Figure 4E–4G). To control for off-target effects of the TRPM7 shRNA, we confirmed the effect of TRPM7 knockdown on SH-EP2 cell migration using cells that were transduced with a second, independent TRPM7 targeting shRNA (Supplementary Figure S1D, Supplementary Figure S2C–S2E). Overall, we conclude that TRPM7 drives neuroblastoma cell migration.

### TRPM7 maintains neuroblastoma cells in a progenitor state

**Figure 5:**
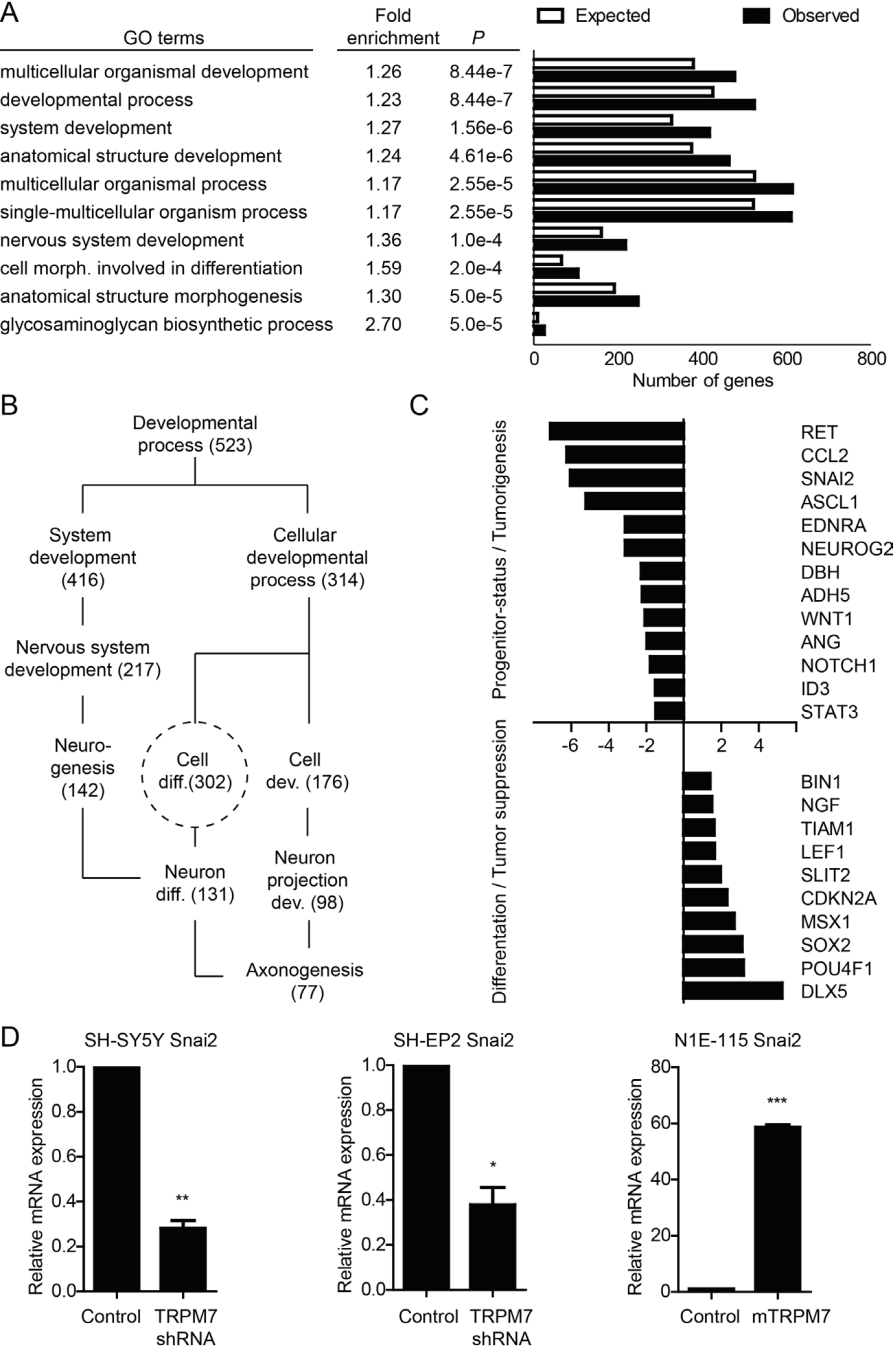
TRPM7 controls progenitor-like features of neuroblastic neuroblastoma cells at the gene expression level **(A)** TRPM7 controls developmental gene expression programs. GO-term analysis was performed on genes that were differentially expressed (*p* < 0.05) in SH-SY5Y TRPM7 shRNA cells, when compared to SH-SY5Y control cells. Presented are the 10 most significantly enriched (*P)* GO-terms within the subroot ‘Biological Process’. Fold enrichment is the ratio between observed and expected genes within a category. Number of expected genes is based on the number of protein encoding genes within a category. **(B)** TRPM7 shRNA affects genes that control neuronal differentiation. Simplified representation of GO-term relationships involved in neuronal differentiation, and the number of genes observed per category. **(C)** TRPM7 shRNA impairs progenitor-status and tumorigenicity of neuroblastic SH-SY5Y neuroblastoma cells. Selection of differentially regulated genes within the ‘Cell Differentiation’ category that are known to control neural crest formation, delamination and differentiation, and neuroblastoma progression (Table 1). X-axis represents fold difference in normalized expression levels between control and TRPM7 shRNA SH-SY5Y cells. **(D)** TRPM7 drives expression of SNAI2 transcription factor in human and mouse neuroblastoma cell lines. SNAI2 expression levels were determined by quantitative RT-PCR in SH-SY5Y and SH-EP2 control and TRPM7 shRNA cells. Additionally, SNAI2 expression levels were quantified in N1E-115 mouse neuroblastoma control and TRPM7 overexpressing cells. Data represents mean expression levels ± SEM (*n* = 3) that are normalized to GAPDH housekeeping gene expression. SNAI2 expression in control cells is set to 1. *p < 0.05, **p < 0.01, ***p < 0.001.

Since TRPM7 was shown to be required for the development of neural crest derived tissues by maintaining the pool of pluripotent progenitor cells [37], we hypothesized that TRPM7 may contribute to neuroblastoma metastasis by preserving progenitor-like, migratory neural crest cell features. We performed microarray-based gene expression profiling and identified 3015 genes that were significantly (*p* < 0, 05) up (1418 genes) or down (1597 genes) regulated in neuroblastic SH-SY5Y TRPM7 shRNA cells, when compared to control cells (http://www.ncbi.nlm.nih.gov/geo/query/acc.cgi?acc=GSE64000). Subsequent Gene Ontology (GO)-term analysis revealed 40 GO categories within the ‘Biological Process’ subroot that were significantly enriched (*p* < 0.01, > 10 genes per category) (Supplementary Table S1). Consistent with a role for TRPM7 in embryonic development [36, 37], GO categories comprising organism and cellular development were most significantly enriched (Figure 5A). Moreover, a simplified graphical representation of relationships between all 40 enriched GO-terms based on the directed acyclic graph as presented by Webgestalt, clearly indicates that genes affected by TRPM7 shRNA are involved in neuronal differentiation (Figure 5B). Within the GO category ‘Cell Differentiation’, we found many genes that are well established to control neural crest development as well as neuroblastoma progression. In view of the complex crosstalk between these genes and the different roles they play at distinct stages of development, it is difficult to define in detail how the observed changes in gene expression affect neuroblastoma progression. In general, genes that promote neural crest development, such as *DLX5*, *LEF1* and *MSX1* were upregulated, whereas genes that are expressed by precursor cells or suppress differentiation, including *ASCL1*, *ID3, SNAI2* and *STAT3*, were down regulated (Figure 5C & Table 1). Hence, TRPM7 expression appears to be inversely associated with neural crest differentiation. Moreover, genes that have previously been established to drive neuroblastoma progression and migration, including *DBH*, *NOTCH1*, *RET* and *WNT1*, were down regulated in response to TRPM7 knockdown (Figure 5C & Table 1). Combined with our *in vitro* and *in vivo* data, these data support the notion that shRNA-mediated knockdown of TRPM7 impairs the malignant potential of human SH-SY5Y neuroblastoma cells at the gene expression level, by reducing a progenitor-like state.

**Table 1:** TRPM7 shRNA impairs progenitor-status and tumorigenicity of neuroblastic SH-SY5Y neuroblastoma cells on the gene expression level Selection of up and down regulated genes in SH-SY5Y TRPM7 shRNA cells (Figure 5B & 5C) that are known to control neural crest differentiation (Diff), migration (Migr) and/or neuroblastoma progression (NB). + positively associated, – negatively associated. (–) or (+) associated with migration or cancer progression, but no direct link with neural crest development or neuroblastoma progression.

	Gene Symbol	Diff.	Migr.	NB.	Description	Ref.
Down	ADH5	–			Negative regulator of neuronal differentiation	(66)
	ANG		(+)	+	Marker for high-risk NB; Induces cancer cell proliferation and migration	(67–69)
	ASCL1	–		+	Marker for high-risk NB; Marker for neuronal precursor cells; Down regulated during NB differentiation	(70–73)
	CCl2		+		Promotes neuronal cell migration	(74)
	DBH	–		+	Marker for high-risk NB; asscociated with impaired terminal differentiation of sympathetic neurons	(75–78)
	EDNRA	–			Edn1-EDNRA signaling suppress neural crest differentiation	(79)
	ID3	–			Maintains undifferentiated state of NC cells	(80)
	NEUROG2	–	+		Essential for neuronal cell migration; Negative regulator of neuronal differentiation	(81, 82)
	NOTCH1	–		+	Marker for high-risk NB; Blocks NB cell differentiation	(83, 84)
	RET	–	+	+	Marker for neuronal precursor cells; Required for NC migration; Promotes neuroblastoma tumorigenesis	(85–88)
	SNAI2	–	+	+	Promotes NC formation; Promotes NC/NB cell migration and invasion; Reduces NC/NB cell apoptosis	(6, 11, 54)
	STAT3	–		+	Maintains undifferentiated state of NC cells; Preserves progenitor-like features of NB cells	(89, 90)
	WNT1	–		+	Maintains undifferentiated state of NC cells; Marker for high-risk NB	(91–93)
Up	BIN1	+		–	Tumor suppressor; Downregulated in high-risk NB; Promotes NB cell differentiation and apoptosis	(94–96)
	CDKN2A			–	Tumor suppressor; Mutated in NB (inactivation); Essential for cell cycle control	(97)
	DLX5	+			Early NC marker; Promotes neuronal differentiation	(98, 99)
	LEF1	+			Induces NC differentiation	(100)
	MSX1	+		–	Early NC marker; Inhibits cell growth and decreases anoikis resistence; Promotes neuronal differentiation	(54, 101)
	NGF	+		–	Induces NB differentiation	(102)
	POU4F1	+			Induces NC differentiation	(103)
	SOX2	+	–		Required for NC formation, proliferation and differentiation; Down regulated during NC delamination and migration	(104)
	SLIT2		–	(–)	Tumor suppressor; Impairs NC/NB cell migration; Impairs glioma metastasis	(105–107)
	TIAM			–	Mutated in high-risk NB (inactivation)	(28)

It seems plausible that TRPM7 shRNA impairs migration of neuroblastic SH-SY5Y by affecting genes that control neuronal precursor characteristics. Despite the lack of neuronal features, TRPM7 shRNA similarly impaired migration of epithelial-like SH-EP2 cells (Figure 4). This suggests that TRPM7-mediated effects on gene expression are not restricted to neuronal cells. We set out to define the transcriptional program controlled by TRPM7 in SH-EP2 cells using a similar approach as described for SH-SY5Y cells. We identified a set of 3322 genes that was differentially expressed by SH-EP2 cells upon TRPM7 knockdown (1664 up, 1658 down, *p* < 0.05). Consistent with the large differences between neuroblastic and epithelial-like neuroblastoma cells, this set of genes showed only modest overlap with the genes that were up or down regulated by TRPM7 shRNA in SH-SY5Y cells (5.6% up, 7.1% down) (Supplementary Figure S3B). GO-term analysis yielded 40 significantly enriched categories within the ‘Biological Process’ subroot (*p* < 0.01, > 10 genes per category), comprising a number of developmental categories that were similar to those identified in SH-SY5Y cells (Supplementary Table S2). However, unlike in the SH-SY5Y cells, neurogenesis-related categories were not found enriched in the SH-EP2 cells. Instead, many GO-terms linked to cell migration were enriched in these cells. Moreover, shRNA-mediated knockdown of TRPM7 in SH-EP2 cells affected expression of genes involved in EMT, a developmental transcription program that allows epithelial cells to become migratory (Supplementary Figure S3A). Transcriptional activators that contribute to EMT, including *SNAI1*, *SNAI2*, *TWIST1*, *HIF1A* and *LEF1*, were significantly down regulated by TRPM7 knockdown, consistent with reduced migration of SH-EP2 TRPM7 shRNA cells. Despite the up regulation of some other EMT inducers such as *SOX9* and *EOMES* upon TRPM7 knockdown, these results suggest that TRPM7 maintains epithelial-like neuroblastoma cells in a progenitor-like migratory state.

In search of transcription factors that are directly controlled by TRPM7, we screened for transcriptional regulators that were affected in both SH-EP2 and SH-SY5Y TRPM7 shRNA cells (Supplementary Figure S3C). In addition to *LIN28B*, *POU4F1*, *ID3* and *SNAI1*, *SNAI2* was of particular interest since its expression was most strongly reduced in both cell lines upon TRPM7 knockdown. Indeed, we confirmed by quantitative RT-PCR that expression of SNAI2 was reduced upon TRPM7 knockdown in both SH-SY5Y and SH-EP2 neuroblastoma cells (Figure 5D & Supplementary Figure S2F). Moreover, overexpression of TRPM7 in the SNAI2 negative N1E-115 mouse neuroblastoma cell line induced expression of SNAI2. Since SNAI2 is not only an important driver of neural crest formation and migration [6, 10, 43], but also a determinant of cancer stemness, migration and metastasis [4, 10, 11, 44], our results indicate that TRPM7 maintains progenitor features of neuroblastoma cells by controlling gene expression programs that involve the transcription factor SNAI2.

## DISCUSSION

Studies using *in vitro* and *in vivo* approaches have identified TRPM7 as a critical regulator of embryogenesis and tissue homeostasis. Deregulation or dysfunction of TRPM7 is associated with a number of pathologies, including cancer [35, 45]. Despite the increasing number of studies on the role of TRPM7 in cancer, data that support the association between TRPM7 expression levels and disease progression in patients are limited. TRPM7 expression levels are increased in nasopharyngeal carcinomas [46] and pancreatic ductal adenocarcinomas [47], and we showed in two independent cohorts that high TRPM7 expression associates with poor prognosis of breast cancer patients at time of diagnosis [34]. In a recent publication, Zhang et. al. observed a strong correlation between TRPM7 and MYCN expression levels in a large neuroblastoma patient cohort (Kocak, *n* = 649) [40]. We were able to confirm a positive association between TRPM7 and MYCN mRNA expression in two out of five additional neuroblastoma patients datasets that are publically available (Supplementary Table S3). However, neither in the Kocak dataset nor in the other datasets did TRPM7 expression associate with neuroblastoma disease stage, an important prognostic marker. In only one of the datasets we found TRPM7 to be associated with relapse, but not with overall survival. Thus, increased TRPM7 expression may correlate with MYCN expression, but whether TRPM7 mRNA expression associates with disease progression in neuroblastoma patients remains to be established.

In this study, we show that TRPM7 overexpression confers a malignant phenotype onto poorly metastatic neuroblastoma cells *in vivo*. This is in close agreement with our recent observations in breast cancer cells, showing that TRPM7 shRNA-mediated knockdown impairs breast cancer metastasis formation in a mouse model [34]. Together, these experiments indicate that high TRPM7 expression levels promote tumor metastasis formation *in vivo*. A number of studies have suggested that TRPM7 contributes to tumor progression by enhancing cell proliferation [40, 48–51]. Using neuroblastoma cells that are made to express high levels of MYCN, experiments by Penner and colleagues suggest that TRPM7 expression is required for MYCN-enhanced proliferation of neuroblastoma cells [40]. However, using neuroblastoma cells that express more physiological levels of MYCN, we find that TRPM7 knockdown and overexpression do not affect proliferation *in vitro*, irrespective of MYCN amplification status. Moreover, we find an increased number of tumors in mice injected with mouse neuroblastoma cells that overexpress TRPM7, rather than increased tumor size. Differences in experimental approach may explain the discordance between our observation and those reported in literature. Whereas we stably overexpressed mTRPM7 or TRPM7 shRNAs by retro- and lentiviral transductions, and subsequently selected viable cells, others manipulated TRPM7 protein levels by transient expression of siRNA oligos. In our hands, transient knockdown as well as overexpression of TRPM7 induces cell death within 48 hours after transfection (data not shown). In agreement, long term exposure to high concentrations of TRPM7 channel blockers reduces cell viability [48, 52, 53]. We propose that by generating stable TRPM7 overexpression and knockdown cell lines, we have modulated TRPM7 expression levels within a range compatible with normal cell viability and proliferation.

High-risk metastatic neuroblastoma cells adopt features used by neural crest progenitor cells to delaminate from the neural border and to colonize different parts of the embryo [4]. Using conditional knockout mice, TRPM7 was found to be required for the maintenance of multi-potent neural crest cells during embryogenesis [37]. Hence, TRPM7 may drive neuroblastoma metastasis by preserving neural crest-like progenitor features. Indeed, we observed that TRPM7 expression is required for migration of neuroblastic (N-type) and epithelial-like (S-type) neuroblastoma cells, resembling the migratory potential of neural crest cells [8]. Using microarray-based expression profiling, we show that TRPM7 controls expression of genes that regulate neural crest development (Figure 5 & Supplementary Figure S3). Gene ontology analysis suggests that TRPM7 maintains an undifferentiated state in N-type SH-SY5Y cells by controlling the expression of neural plate border and neural crest specifiers, whereas it preserves a motile phenotype in S-type SH-EP2 cells by controlling genes that drive the epithelial-to-mesenchymal transition. It should be noted, however, that genes controlling neural crest development play distinct roles at different stages of development. Attributing genes a specific role in neural crest development, i.e. neural plate border and neural crest specification, induction of migration (EMT) and late differentiation, is an oversimplification of the complex crosstalk between genes during development [6]. However, based on an extensive literature search (Table 1), we postulate that TRPM7 knockdown redirects neuroblastoma gene expression by reducing stemness and motility, and promoting differentiation.

The limited overlap in genes that are controlled by TRPM7 in the two cell models is reminiscent of the concept that signaling inputs and transcription factors involved in neural crest development act as time and context dependent gene regulatory networks [6]. Since SNAI2 function is crucial to early neural crest specification, as well as EMT-driven delamination and survival of neural crest cells outside of the niche, down regulation of SNAI2 expression may underlie the distinct effects on developmental gene expression programs upon TRPM7 shRNA in N-type and S-type neuroblastoma cells [6, 10, 43, 44, 54].

Consistent with its function in neural crest development [44], SNAI2 is an important driver of cancer progression. SNAI2 expression is suggested to maintain the pool of cancer stem cells, allowing tumor cells to leave the primary tumor, colonize ectopic tissues, and induce therapy resistance [44, 55, 56]. Moreover, SNAI2 drives EMT in neuroblastoma cells [10], and, reciprocally, SNAI2 knockdown increases the sensitivity to apoptosis-inducing compounds and impairs the metastatic potential of neuroblastoma cells *in vitro* and *in vivo* [11]. Hence, our results strongly suggest that TRPM7 maintains stem cell features of neuroblastoma cells by expression regulation of SNAI2.

Although the findings presented in this study are in line with the accumulating evidence that TRPM7 plays a crucial role during embryogenesis, cellular differentiation and overall tissue homeostasis [35, 45], the precise signaling mechanisms and transcriptional regulators involved remain largely elusive. Supporting the idea that developmental processes are controlled in a context-dependent manner, TRPM7 activity is responsive to the physical characteristics of the microenvironment [57, 58] and modulates mesenchymal stem cell differentiation upon mechanical stimulation [59]. In addition to activating Ca^2+^-dependent transcription factors upon mechanical stimulation, including STAT3 and NFATC1 [37, 59–61], TRPM7 may translate mechanical cues from the microenvironment into gene expression alternations by modulating cytoskeletal tension. Actomyosin-driven cytoskeletal tension is an important determinant of neural crest formation [62]. For instance, reduced cytoskeletal tension promotes the expression of neural crest specifiers including SNAI2. Consistent with the association between TRPM7 and SNAI2 expression levels in our neuroblastoma cell models, we previously showed that TRPM7 activity induces cytoskeletal relaxation in breast cancer cells as well as in neuroblastoma cells [33, 34]. Although the mechanism by which TRPM7 affects gene expression remains to be explored, our results suggest a model in which context dependent activation of TRPM7 reduces cytoskeletal tension, and through activation of transcriptional regulators that respond to cytoskeletal dynamics, such as YAP/TAZ and SRF [63, 64], controls the malignant features of neuroblastoma cells by promoting neural crest stem cell properties. Future studies will determine how alterations in cytoskeletal dynamics affect the expression of SNAI2, and whether TRPM7-driven SNAI2 expression contributes to neuroblastoma progenitor-like features. Ultimately, specific TRPM7 inhibitors such as Waixenicin A [48], could potentially be used to induce neuroblastoma differentiation, and may prove useful in treatment of neuroblastoma in future (pre-) clinical studies.

## MATERIALS AND METHODS

### Constructs and cell lines

N1E-115 and SH-EP2 cells were cultured in DMEM supplemented with 10% FCS and 1% penicillin-streptomycin, at 37°C and 5% CO_2_. SH-SY5Y were maintained in DMEM/F12 medium supplemented with 10% FCS, 1% penicillin-streptomycin, 1% non-essential amino acids and 1% L-glutamine.

Cloning of full length TRPM7-HA cDNA into LZRS-neo was previously described [33]. Luciferase cDNA was isolated from pMX-luciferase-YFP-neo and subcloned into a retroviral pLZRS-IRES-zeocin vector. N1E-115 mouse and SH-SY5Y human neuroblastoma cells stably overexpressing TRPM7-HA and empty vector control were generated by retroviral transductions. Transduced cells were selected by the addition of 1 mg/ml G418. For bioluminescent imaging, control and TRPM7 overexpressing N1E-115 cells were co-transduced with a retroviral pLZRS luciferase reporter construct and selected with 0.5 mg/ml Zeocin. Human TRPM7 shRNA (5-GCGCTTTCCTTATCCACTTAA-3) was introduced in SH-SY5Y (CRL-2266, ATCC) and SH-EP2 (J. Molenaar, AMC, Amsterdam, The Netherlands) cells, using the pLKO lentiviral expression vector according to manufacturer's instructions (Sigma Aldrich, St. Louis, MO). An independent TRPM7 shRNA was introduced in SH-EP2 to control for off-target effects (TRPM7 shRNA#2: 5-TTGCCTGTAAGATCTATCGTT-3). A nonfunctional shRNA (5-GCTACAAGAGAAACCAAATCT-3) was introduced in SH-SY5Y and SH-EP2 cells to serve as negative control. Transduced cells were selected with 1 μg/ml puromycin.

The effect of TRPM7 overexpression and knockdown on cell viability and proliferation was assessed by MTS assays according to manufacturer's instructions (Promega, Madison, WI).

### Quantitative RT-PCR

Total mRNA isolation (Qiagen, Valencia, CA) was followed by SuperScript cDNA synthesis (Life Technologies, Carlsbad, CA), according to manufacturer's protocols. TRPM7 mRNA expression levels in N1E-115, SH-EP2 and SH-SY5Y cells were determined by quantitative PCR reactions using *power SYBR-green* reagent (Applied Biosystems, Carlsbad, CA) in combination with mouse specific (forward: TAGCCTTTAGCCACTGGACC; reverse: GCATCTTCTCCTAGATTGGCAG) or human specific (forward: TAGCCTTTAGCCACTGGAC; reverse: GCATCTTCTCCTAGATTTGC) TRPM7 primers according to manufacturer's recommendations. Using the similar approach, SNAI2 levels were determined using mouse specific (forward: GGCTGCTTCAAGGACACATT; reverse: GGTTTTGGAGCAGTTTTTGC) and human specific (forward: AGATGAGCATTGGCAGCGAG; reverse: AAGCATTTCAACGCCTCCAAA) SNAI2 primers. Mouse and human TRPM7 and SNAI2 mRNA expression levels were normalized to the mouse (forward: GCCAAGGTCATCCATGACAAC; reverse: GAGGGGCCATCCACAGTCTT) or human (forward: CTCCTCCACCTTTGACGCTG; reverse: TCCACCACCCTGTTGCTGTA) GAPDH housekeeping genes respectively, and calculated according to the cycling threshold method [65].

### Cell migration assays

Following overnight serum starvation, cells were harvested and resuspended in DMEM containing 0.1% FBS. Subsequently, 50.000 cells were applied to a transwell insert with 8 μm pore size (Corning Life Sciences, Corning, NY), which was incubated in DMEM supplemented with 10% FBS. N1E-115 and SH-SY5Y cells were allowed to migrate for 48 hours at 37°C towards a serum gradient. SH-EP2 cells were allowed to migrate for 24 hours. Migrated cells were fixed (75% methanol and 25% acetic acid) and stained (0.25% Coomassie Blue, 45% methanol, 10% acetic acid in H_2_O). Gap closure assays were performed according to manufacturer's recommendations (Applied Biophysics, Troy, NY). In short, 50.000 SH-EP2 cells were seeded per insert and cultured overnight. After removal of the insert, cells were allowed to migrate for 24 hours and migration was followed by time lapse microscopy for 24 hours. Gap closure speed was quantified using ImageJ (version 1.48) image analysis software

### Mouse xenograft experiments

All animal work was performed in accordance with protocols approved by the Animal Welfare Committee (DEC-NKI-10.034). *Rag2^−/−^*Il2rg*^−/−^* immunodeficient mice, backcrossed on a Balb/c background, were used for metastasis experiments at 5–8 weeks old. N1E-115 mouse neuroblastoma cells were trypsinized and washed 3 times with PBS. Subsequently, 0.2 ml PBS containing 5*10^5^ cells was injected into a tail vein. Tumor growth was monitored by bioluminescence imaging from day 7 onwards. Beetle luciferin (Promega, Fitchburg, WI, USA) was dissolved at 15 mg/ml in sterile PBS and stored at –20°C. Animals were anaesthetized with 2–3% isoflurane. Luciferin solution was injected i.p. (0.01 ml per gram body weight). Light emission was measured 15 min later, using a cooled CCD camera (IVIS; Xenogen), coupled to Living Image acquisition and analysis software over an integration time of 1 min. Signal intensity was quantified as the Flux (photons / s) measured over the abdominal region. Organs and tissues were collected at day 20 after injection, fixed in EAF (ethanol-acetic acid-formol saline fixative, 40:5:10:45 v/v) and processed routinely for histology preparations. The paraffin sections were stained with Haematoxylin and Eosin (H&E). For quantitative analysis of the neoplastic lesions, liver sections (9 sections from each liver) were stained with Haematoxylin and Eosin. Liver tumors were quantified under a microscope and the data were further processed by Image J (version 1.48).

### Microarray

Microarray-based gene expression profiling on SH-SY5Y and SH-EP2 control and shTRPM7 cells was performed by ServiceXS B.V. (Leiden, The Netherlands). Samples were prepared and analyzed in duplo. mRNA was purified from cell cultures and treated with DNAse (Qiagen, Valencia, CA). RNA concentrations were measured using the Nanodrop ND-1000 spectrophotometer (Nanodrop Technologies, Wilmington, DE. U.S.A). RNA quality and integrity were determined using Lab-on-Chip analysis on the Agilent 2100 Bioanalyzer (Agilent Technologies, Inc., Santa Clara, CA, U.S.A). Biotinylated cRNA was prepared using the Illumina TotalPrep RNA Amplification Kit (Ambion, Inc., Austin, TX, U.S.A) according to manufacturer's specifications with an input of 200 ng total RNA. Per sample, 750 ng of the obtained biotinylated cRNA samples was hybridized onto the Illumina HumanHT-12 v4 (Illumina, Inc., San Diego, CA, U.S.A). Hybridization and washing were performed according to the Illumina Manual ‘Direct Hybridization Assay Guide’. Scanning was performed on the Illumina iScan (Illumina, Inc., San Diego, CA, U.S.A). Image analysis and extraction of raw expression data was performed with Illumina GenomeStudio v2011.1 Gene Expression software. Arrays were normalized in Arraystar (v. 4.03), using Robust-Multi-Array normalization. Fold difference and *P*-values were calculated using Multi Experiment Viewer (v. 4.8.1). GO-term analysis was performed using Webgestalt (http://bioinfo.vanderbilt.edu/webgestalt/) on genes that were significantly (*p* < 0.05) up or down regulated. Vennmaster (http://sysbio.uni-ulm.de/?Software:VennMaster) was used to determined the overlap in genes that were significantly up or down regulated in SH-SY5Y and SH-EP2 TRPM7 shRNA cells.

## SUPPLEMENTARY FIGURES AND TABLES


