# Supplementary material for: TRPM7 maintains progenitor-like features of neuroblastoma cells: implications for metastasis formation
Source: Oncotarget. 2015 Mar 5;6(11):8760–76. doi: 10.18632/oncotarget.3315 (PMC4496182; doi:10.18632/oncotarget.3315)
Supplement: Supplementary file 1 [file oncotarget-06-8760-s001.pdf]

SUPPLEMENTARY FIGURES AND TABLES

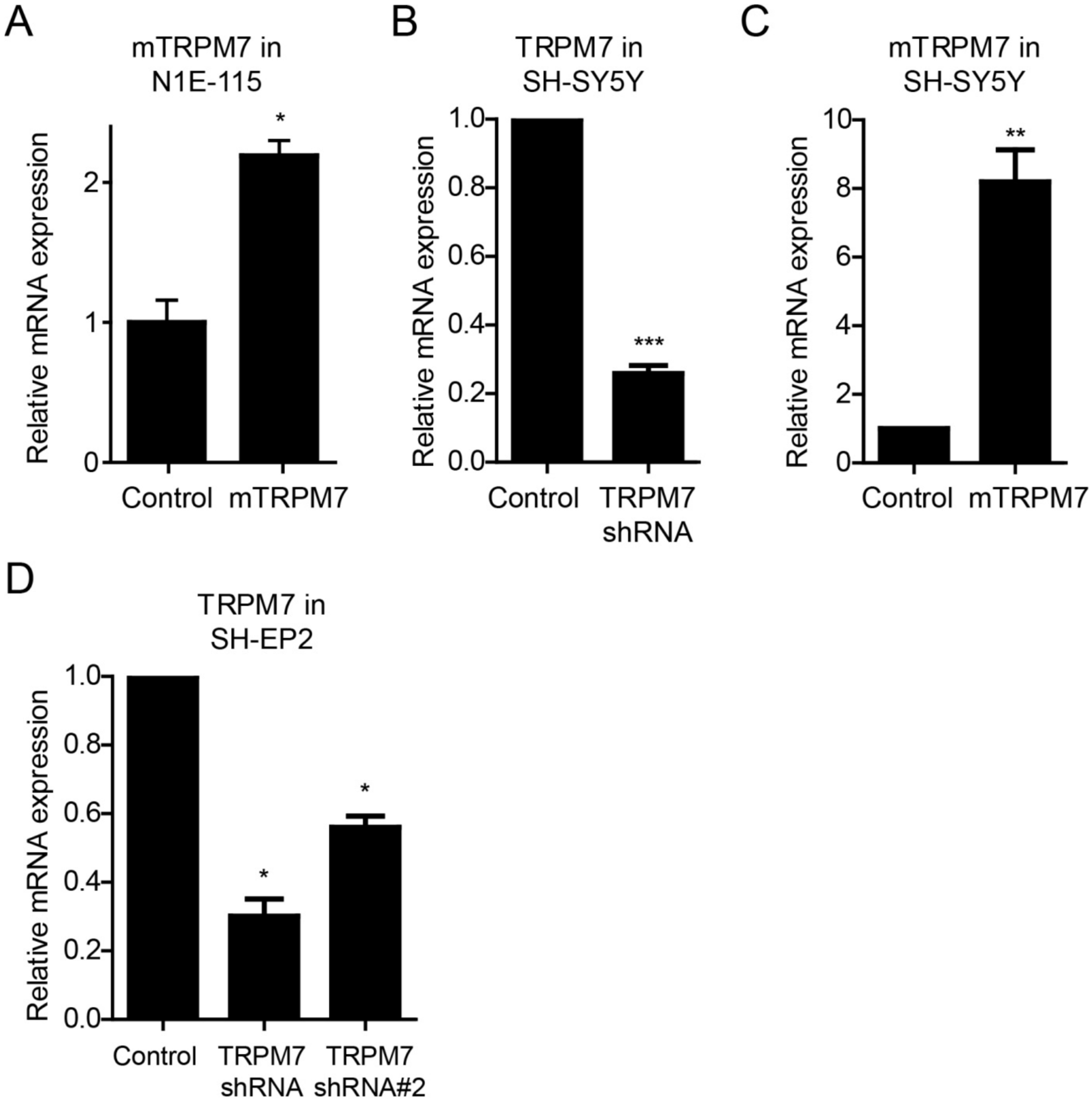

Supplementary Figure S1: Manipulation of TRPM7 expression in mouse N1E-115 cells, and human SH-SY5Y and SH-EP2 cells. (A) Mouse TRPM7 mRNA expression in mouse N1E-115 control and mTRPM7 overexpressing cells, determined by quantitative RT-PCR. Mouse TRPM7 expression in the control cells is set to one. Data is mean ± SEM of $n = 2$ independent experiments. **(B)** Human TRPM7 mRNA expression in human SH-SY5Y control and TRPM7 shRNA cells, determined by quantitative RT-PCR. Human TRPM7 expression in the control cells is set to one. Data is mean ± SEM of $n = 6$ independent experiments. **(C)** Mouse TRPM7 mRNA expression in human SH-SY5Y control and mTRPM7 overexpressing cells, determined by quantitative RT-PCR. Mouse TRPM7 expression in the control cells is set to one. Data is mean ± SEM of $n = 3$ independent experiments. **(D)** Human TRPM7 mRNA expression in human SH-EP2 control, TRPM7 shRNA and TRPM7 shRNA#2 cells, determined by quantitative RT-PCR. Human TRPM7 expression in the control cells is set to one. Data is mean ± SEM of $n = 2$ independent experiments. *$p < 0.05$, **$p < 0.01$, ***$p < 0.001$.

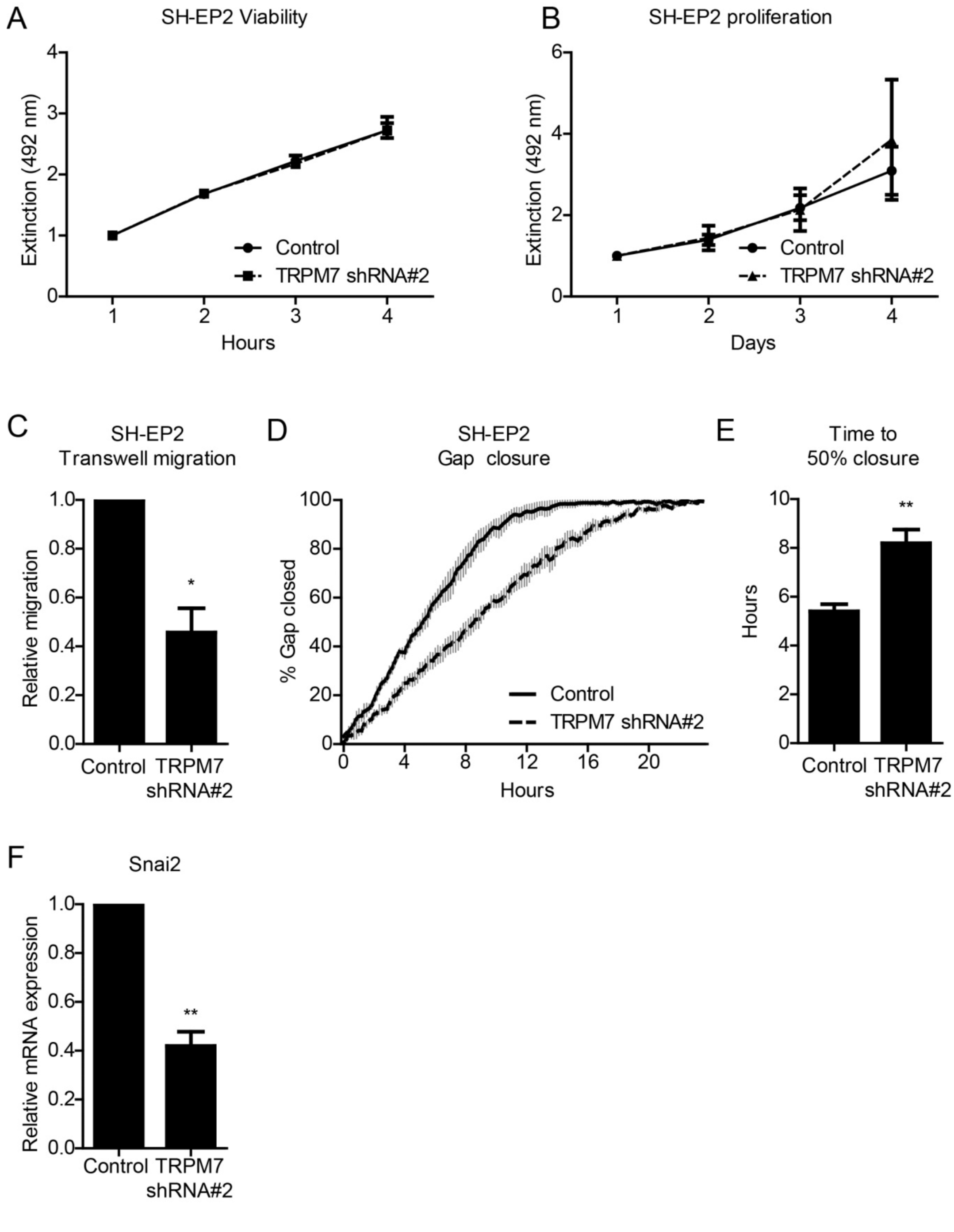

Supplementary Figure S2: TRPM7 shRNA#2 reduces SH-EP2 cell migration and SNAI2 mRNA expression, but not cell viability and proliferation. **(A)** & **(B)** Effect of TRPM7 shRNA#2 on viability and proliferation of SH-EP2 cells. Quantification of cell viability and proliferation, determined by MTS assays. Viability was assessed over 4 hours, proliferation was assessed over 4 days. Data represents normalized mean extinction at 492 nm mean ± SEM of $n = 2$ experiments performed in triplo. **(C)** Transwell migration of SH-EP2 control and TRPM7 shRNA#2 cells. Equal numbers of cells were allowed to migrate towards a serum gradient for 24 hours. Data are normalized to control and represent mean ± SEM of $n = 3$ independent experiments performed in duplicate. **(D)** Gap closure over time, presented as percentage of gap size at time point 0 hours. **(E)** Quantification of time to 50% gap closure. Data in (D) and (E) represent mean ± SEM from $n = 3$ independent experiments, each performed in duplicate. **(F)** TRPM7 shRNA#2 reduces SNAI2 mRNA expression levels in SH-EP2 cells, as determined by quantitative RT-PCR. Data represents mean expression levels ± SEM ($n = 3$) that are normalized to GAPDH housekeeping gene expression. SNAI2 expression in control cells is set to one. *$p < 0.05$, **$p < 0.01$, two-tailed unpaired t-test.

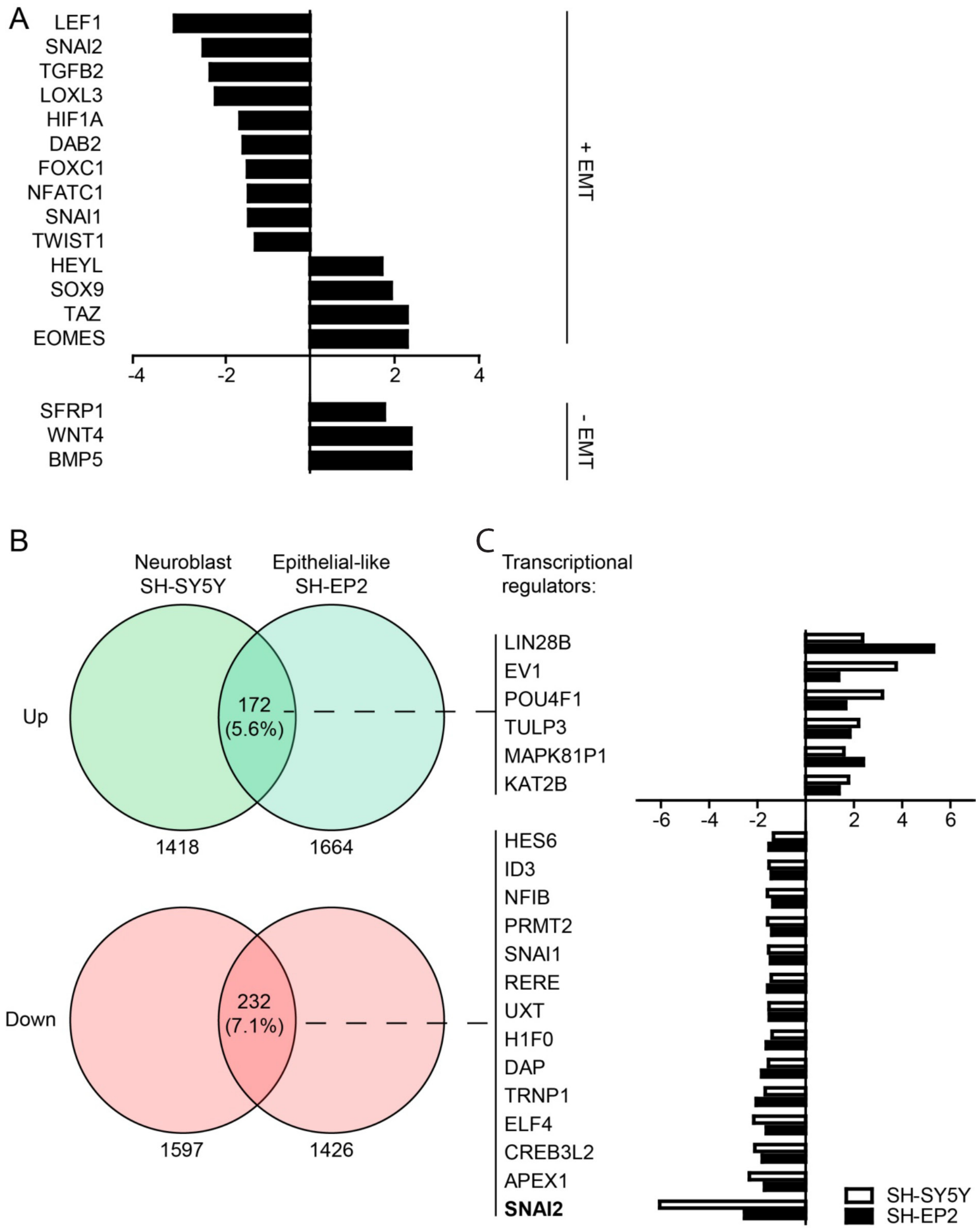

Supplementary Figure S3: TRPM7 shRNA reduces expression of EMT transcription factors in SH-EP2 cells. **(A)** TRPM7 shRNA induces a partial mesenchymal to epithelial transition in SH-EP2 human neuroblastoma cells. Selection of differentially regulated genes within the 'Epithelial to mesenchymal transition' category, that are known to positively (+) or negatively (–) associate with epithelial to mesenchymal transition in neural crest and neuroblastoma cells. X-axis represents fold difference in normalized expression levels between control and TRPM7 shRNA cells. **(B)** Overlap in differentially expressed genes in neuroblastic SH-SY5Y and epithelial-like SH-EP2 cells. **(C)** Transcriptional regulators that are up or down regulated in both neuroblastic SH-SY5Y and epithelial-like SH-EP2 cells. X-axis represents fold difference in normalized expression levels between control and TRPM7 shRNA cells.

Supplementary Table S1: GO-term analysis on TRPM7 shRNA affected genes in SH-SY5Y cells

GO-term analysis using Webgestalt yields 40 significantly enriched categories (*P*). Fold enrichment is the ratio between observed and expected genes within a category. Number of expected genes is based on the number of protein encoding genes within a category.

GO-term	Observed	Expected	Fold enrichment	*P*
multicellular organismal development	476	378.16	1.26	8.44e-07
developmental process	523	424.07	1.23	8.44e-07
system development	416	326.59	1.27	1.56e-06
anatomical structure development	463	373.80	1.24	4.61e-06
multicellular organismal process	613	523.51	1.17	2.55e-05
single-multicellular organism process	610	520.54	1.17	2.55e-05
nervous system development	217	159.91	1.36	0.0001
cell morphogenesis involved in differentiation	104	65.30	1.59	0.0002
anatomical structure morphogenesis	247	190.61	1.30	0.0005
glycosaminoglycan biosynthetic process	25	9.28	2.70	0.0005
aminoglycan biosynthetic process	25	9.37	2.67	0.0005
blood vessel development	76	45.64	1.67	0.0006
tissue development	182	134.40	1.35	0.0007
chondroitin sulfate proteoglycan metabolic process	17	5.19	3.27	0.0007
neuron projection morphogenesis	86	54.08	1.59	0.0008
anatomical structure formation involved in morphogenesis	196	147.85	1.33	0.0011
neuron differentiation	131	91.83	1.43	0.0011
cell morphogenesis involved in neuron differentiation	84	53.33	1.57	0.0012
chondroitin sulfate metabolic process	16	5.01	3.19	0.0013
cell proliferation	190	143.58	1.32	0.0013
cell adhesion	126	88.49	1.42	0.0014
biological adhesion	126	88.67	1.42	0.0015
cell-cell adhesion	63	37.47	1.68	0.0015
neuron projection development	98	65.30	1.50	0.0015
vasculature development	76	47.95	1.58	0.0017
axonogenesis	77	48.79	1.58	0.0017
organ development	291	236.71	1.23	0.0019
cell differentiation	302	247.19	1.22	0.0020
response to chemical stimulus	310	254.70	1.22	0.0020
cell-cell junction organization	26	11.50	2.26	0.0025
spinal cord motor neuron differentiation	10	2.41	4.15	0.0026
cell morphogenesis	122	87.65	1.39	0.0030
cell projection organization	120	86.63	1.39	0.0030

(*Continued*)

negative regulation of developmental process	76	49.99	1.52	0.0030
cellular developmental process	314	262.40	1.20	0.0030
cell differentiation in spinal cord	13	3.99	3.26	0.0030
cell part morphogenesis	93	64.09	1.45	0.0030
cell development	176	135.51	1.30	0.0030
neurogenesis	142	105.74	1.34	0.0030
glycosaminoglycan metabolic process	29	13.82	2.10	0.0030

Supplementary Table S2: GO-term analysis on TRPM7 shRNA affected genes in SH-EP2 cells
GO-term analysis using Webgestalt yields 40 significantly enriched categories (P). Fold enrichment is the ratio between observed and expected genes within a category. Number of expected genes is based on the number of protein encoding genes within a category. Categories that were also found enriched in SH-SY5Y TRPM7 shRNA cells (Supplementary Table S1) are indicated in grey.

GO-term	Observed	Expected	Fold enrichment	*P*
response to wounding	183	118.47	1.54	2.59e-07
circulatory system development	135	80.62	1.67	2.59e-07
vasculature development	103	55.28	1.86	2.59e-07
cardiovascular system development	135	80.62	1.67	2.59e-07
cellular component movement	192	126.81	1.51	3.04e-07
blood vessel development	96	52.60	1.82	7.05e-07
blood vessel morphogenesis	86	46.30	1.86	1.74e-06
response to external stimulus	204	141.46	1.44	3.28e-06
renal system development	57	26.94	2.12	4.77e-06
single-multicellular organism process	696	598.64	1.16	8.11e-06
multicellular organismal process	699	602.07	1.16	8.58e-06
response to lipid	104	62.23	1.67	9.64e-06
urogenital system development	62	31.22	1.99	9.89e-06
regulation of response to external stimulus	74	40.20	1.84	1.42e-05
angiogenesis	71	38.17	1.86	1.56e-05
cell migration	137	89.81	1.53	1.94e-05
wound healing	108	66.93	1.61	2.41e-05
locomotion	188	133.01	1.41	2.49e-05
cell motility	145	97.19	1.49	2.62e-05
localization of cell	145	97.19	1.49	2.62e-05
system development	456	376.04	1.21	3.59e-05
anatomical structure morphogenesis	285	219.61	1.30	4.32e-05
response to organic cyclic compound	100	61.91	1.62	4.74e-05
single–organism process	904	815.69	1.11	6.43e-05

(*Continued*)

kidney development	46	22.24	2.07	7.23e-05
developmental process	570	487.66	1.17	8.14e-05
response to molecule of bacterial origin	48	24.06	2.00	9.64e-05
regulation of cell motility	76	44.59	1.70	9.64e-05
cell adhesion	147	101.68	1.45	9.64e-05
circulatory system process	64	35.50	1.80	9.64e-05
biological adhesion	147	101.89	1.44	9.64e-05
regulation of localization	194	141.78	1.37	9.64e-05
regulation of cell migration	73	42.23	1.73	9.64e-05
epithelial to mesenchymal transition	23	8.13	2.83	0.0001
ERK1 and ERK2 cascade	32	13.79	2.32	0.0001
system process	237	180.91	1.31	0.0001
anatomical structure development	507	430.25	1.18	0.0001
regulation of locomotion	80	47.90	1.67	0.0001
regulation of body fluid levels	100	63.72	1.57	0.0001
blood circulation	63	35.39	1.78	0.0001

Supplementary Table S3: TRPM7 does not correlate with disease stage and progression in neuroblastoma patients

R indicates correlation coefficient. P indicates significance of correlations. Statistical significant correlations ($p < 0.05$), are indicated in red. Analysis on patient datasets were performed using R2 (http://r2.amc.nl).

Dataset	Subcohort	#Patients	TRPM7 vs MYCN mRNA		TRPM7 vs MYCN status		TRPM7 vs Stage		TRPM7 vs Relapse		TRPM7 vs Survival	
			R	P	R	P	R	P	R	P	R	P
Hiyama	total	51	–0.328	0.02	–0.243	0.09	–0.105	0.46			–0.12	0.4
	mycn-neg	44					0.03	0.84			0.012	0.94
	mycn-pos	7					0.143	0.76			0.101	0.83
Jagannathan	total	100	0.259	0.0093	0.055	0.59	–0.09	0.37				
	mycn-neg	78					–0.093	0.42				
	mycn-pos	22					–0.228	0.31				
Kocak	total	649	0.115	0.0035	0.127	0.0012	0.002	0.97				
	mycn-neg	550					–0.025	0.56				
	mycn-pos	93					–0.186	0.07				
	n.s.	6										
Lastowska	total	30	0.181	0.34	0.138	0.47	0.072	0.7				
	mycn-neg	20					0.004	0.99				

(*Continued*)

	mycn-pos	10									
Seeger	total	117	0.299	0.001					0.333	0.00024		
	mycn-neg	n.s.									
	mycn-pos	n.s.									
Versteeg	total	88	–0.06	0.58	0.171	0.11	0.129	0.23	0.056	0.61	0.029	0.79
	mycn-neg	72					0.046	0.7	0.012	0.92	–0.038	0.75
	mycn-pos	16					0.216	0.42	–0.371	0.16	–0.371	0.16
